# Oxytocin use in trial of labor after cesarean and its relationship with risk of uterine rupture in women with one previous cesarean section: a meta-analysis of observational studies

**DOI:** 10.1186/s12884-020-03440-7

**Published:** 2021-01-06

**Authors:** Huan ZHANG, Haiyan LIU, Shouling LUO, Weirong GU

**Affiliations:** Department of Obstetrics and Gynecology, Obstetrics and Gynecology Hospital of Fudan University, Shanghai, 200011 China

**Keywords:** Trial of labor after a previous cesarean delivery, Safety, Oxytocin, Uterine rupture

## Abstract

**Background:**

Trial of labor after a previous cesarean delivery (TOLAC) has reduced the rate of cesarean sections (CS). Nevertheless, the widespread use of TOLAC has been limited by an increase in adverse outcomes, the most serious one being the risk of symptomatic uterine rupture, which is possibly associated with oxytocin. In this meta-analysis, we explored the risk association between oxytocin use and uterine rupture in TOLAC.

**Methods:**

Multiple electronic databases (PubMed, Embase, Web of Science, and Google Scholar) were searched for cross-sectional studies reporting on TOLAC, oxytocin and uterine rupture, which were published between January 1986 and October 2019. The bias-corrected Hedge’s g was calculated as the effect size using the random-effects model. A two-sample Z test was used to compare the differences in synthetic rates between groups. The Newcastle-Ottawa Scale (NOS) was used to evaluate the risk of bias. Quality of the evidence was assessed with the Grading of Recommendations Assessment, Development, and Evaluation (GRADE) certainty ratings system.

**Results:**

A total of 14 studies, which included 48,457 women who underwent TOLAC, met the inclusion criteria. The pooled rate of vaginal birth after a cesarean section (VBAC) and the rate of uterine rupture in spontaneous labor were 74.3 and 0.7%, respectively. In addition, the pooled rate of VBAC and the rate of uterine rupture in the induction labor group was 60.7 and 2.2%, respectively. The women who had spontaneous labor had a significantly higher rate of VBAC (*p* = 0.001) and a lower rate of uterine rupture (*p* = 0.0003) compared to induced labor. The pooled rates of uterine rupture in women using oxytocin and women not using oxytocin in TOLAC were 1.4% and 0.5%, respectively, and the difference was significant (*p* = 0.0002). Also, the synthetic rate of uterine rupture in oxytocin augmentation among women with spontaneous labor and women who had a successful induction of labor were 1.7% and 2.2%, respectively, without significant difference (*p* = 0.443).

**Conclusions:**

Women with induced labor had a higher risk of uterine rupture compared to women with spontaneous labor following TOLAC. Oxytocin use may increase this risk, which could be influenced by the process of induction or individual cervix condition. Consequently, simplified and standardized intrapartum management, precise protocol, and cautious monitoring of oxytocin use in TOLAC are necessary.

**Supplementary Information:**

The online version contains supplementary material available at 10.1186/s12884-020-03440-7.

## Background

Cesarean section (CS) is the most frequently performed surgical procedure in the world. An increasing rate of CS has increased the rate of a uterine scar after surgery [1, 2]. Pregnant women who already underwent CS are usually recommended two delivery options: trial of labor after a previous cesarean delivery (TOLAC) or elective repeat CS. However, repeated CS is associated with an increased risk of morbidity and mortality. On the other hand, TOLAC has fewer complications, and faster recovery compared with CS [3, 4] and provides an option for women who are willing to undergo vaginal birth after a cesarean section (VBAC) [5]. Yet, TOLAC has also been associated with certain maternal and neonatal complications as well as the more stringent criteria [3, 6].

Symptomatic uterine rupture is the most serious complication of TOLAC [7] and an uncommon obstetric emergency. Its reported incidence is approximately 1%, with a range of 0.3–1.7% during the trial of labor [8–10]. In 1995, the American College of Obstetricians and Gynecologists (ACOG) concluded that oxytocin use for induction or augmentation of labor in TOLAC had no contraindications [11]. Flamm et al provided evidence in support of this conclusion [12]. However, some studies reported that the increased risk of uterine rupture might be associated with oxytocin induction or augmentation after controlling for potential confounders [13, 14]. Moreover, other studies have reported an increased rate of uterine rupture associated with either high-dose oxytocin or the use of oxytocin in the latent phase [15, 16]. Also, the administration of oxytocin during the process of labor was found to be related to an increased rate of uterine rupture [17, 18].

This meta-analysis aimed to evaluate the safety of oxytocin in TOLAC and compared the risk of uterine rupture between women using oxytocin and those not using oxytocin during TOLAC, and the risk of uterine rupture between oxytocin augmentation among women with spontaneous labor and those who had a successful induction of labor.

## Method

### Design and registration

We conducted the review according to the registered protocol PROSPERO CRD42020152819 (https://www.crd.york.ac.uk/PROSPERO/) the Preferred Reporting Items for Systematic Reviews and Meta-Analyses (PRISMA) statement.

### Inclusion and exclusion criteria

The inclusion criteria were the following: 1) participants: women with singleton pregnancy after 37 weeks of gestation and a previous low transverse cesarean section (CS), who volunteered and accepted a trial of labor after CS (TOLAC), and did not have any contraindications to TOLAC, such as previous uterine body incision, placenta previa or abnormal pelvimetry with a breech presentation; 2) intervention: oxytocin induction or augmentation during a TOLAC; 3) comparator: women not using oxytocin in TOLAC; 4) outcomes: the success rate of TOLAC (VBAC), the usage rate of oxytocin in TOLAC and the risk of uterine rupture; 5) type of studies: designed with case-control study or cross-sectional study.

The exclusion criteria were: 1) women with previous classical CS, history of more than one CS, major fetal anomalies, active labor before rupturing of membranes, scheduled elective CS, contraindications for spontaneous delivery (placenta previa, breech presentation, etc.), history of uterine rupture, and lack of information regarding the previous delivery; 2) duplication of previous publication(s). Two independent investigators finished the procedure; disagreement was solved by discussion.

### Searching strategies

A literature search of PubMed, EMBASE, Web of Science, Clinical trial, and Google Scholar was conducted with no date restrictions. The following keywords were used: “vaginal birth after a cesarean section” OR “VBAC” AND “a trial of labor after the cesarean section” OR “TOLAC” OR “trial of labor” AND “oxytocin” OR “oxytocin infusion” OR “induction of labor” AND “uterine rupture” OR “maternal morbidity” (see Supplementary materials). Final literature searches were performed in June 2019. The hits were reviewed, and duplicates were eliminated. Then, inclusion and exclusion criteria were set for including records. Finally, the titles, abstracts, keywords, and whole texts of retrieved studies were checked to exclude irrelevant ones. Also, the reference lists of the retrieved studies and recent reviews were manually checked to avoid missing any studies meeting the inclusion criteria.

### Data extraction

Necessary data from eligible studies were extracted in this meta-analysis, including first author’s name, publication year, sample size, maternal age, maternal BMI, gestational age, a dose of oxytocin, the number of spontaneous deliveries, the number of induced labors, the number of patients using oxytocin, the number of VBAC, and the number of uterine ruptures. Uterine rupture was defined as a disruption of the uterine muscle extending to, or involving the uterine serosa, or disruption of the uterine muscle with extension to the bladder or broad ligament (non-reassuring fetal heart rate, abdominal pain, vaginal bleeding, signs of intra-abdominal hemorrhage, hematuria, disengagement of fetal presentation, and signs of maternal shock) [19, 20]. Two independent reviewers performed the double-extraction of the data and cross-checked the results for the discrepancy, which were discussed for correction. A third independent reviewer assessed the coding for accuracy by randomly selecting and recoding five articles and examining potential outliers in the data. The authors were contacted by e-mail and relevant data were requested if these values were not reported so as to collect the complete dataset. In the case of no feedback, the studies with missing information were abandoned.

### Statistical analysis

In order to evaluate the effect of oxytocin, studies were grouped according to those that used oxytocin and those that did not. To explore the factors likely to enhance the effect on uterus, studies were grouped as follows: those reporting on oxytocin augmentation among women with spontaneous labor, and those reporting on women who had successfully induced labor. We calculated the usage rate of oxytocin, rate of VBAC, and rate of uterine rupture by the number of TOLAC, the number of spontaneous delivery, the number of induction labor, the number of VBAC, the number of patients using oxytocin, and the number of uterine ruptures. Due to the anticipated heterogeneity, the random-effects model or fixed-effects model were used to calculate the overall effect size. For each measure, we calculated Cohen’s d in line with the general systematic approach and performed with the associated website (http://www.campbellcollaboration.org/ resources/ effect size input.php), using means and standard deviations or standard errors where possible [21]; occasionally, F, t or *p* values were used with sample size to estimate the effect size. In order to correct for overestimation of the effect size associated with small sample sizes, we applied Hedge’s correction to each effect size, and calculated inverse variance weights for each study using the corrected effect size. Also, we used a two-sample Z test to compare the difference in synthetic rates between groups. A *P* value < 0.05 was considered statistically significant.

Heterogeneity was tested using the I^2^ statistic and Q test. We considered statistical heterogeneity low for I^2^ ≤ 40%, moderate for I^2^ = 30–60%, substantial for I^2^ = 50–90% and considerable for I^2^ = 75–100%. Sensitivity analyses and meta-regression were used to explore the potential sources of heterogeneity. Publication bias was checked using the funnel plot and Egger’s tests. Besides, the critical evaluation of the bias risk of the included studies was conducted by two independent reviewers using the Newcastle-Ottawa Scale (NOS) [22]. In addition, all statistical analyses were conducted using Stata 14.0.

### Assessment of evidence in cumulative evidence

We evaluated the quality of evidence for each outcome across studies using four levels (high, moderate, low or very low confidence) according to the Grading of Recommendations Assessment, Development and Evaluation (GRADE) criteria [23].

## Results

### Characteristics of included studies

**Fig. 1:**
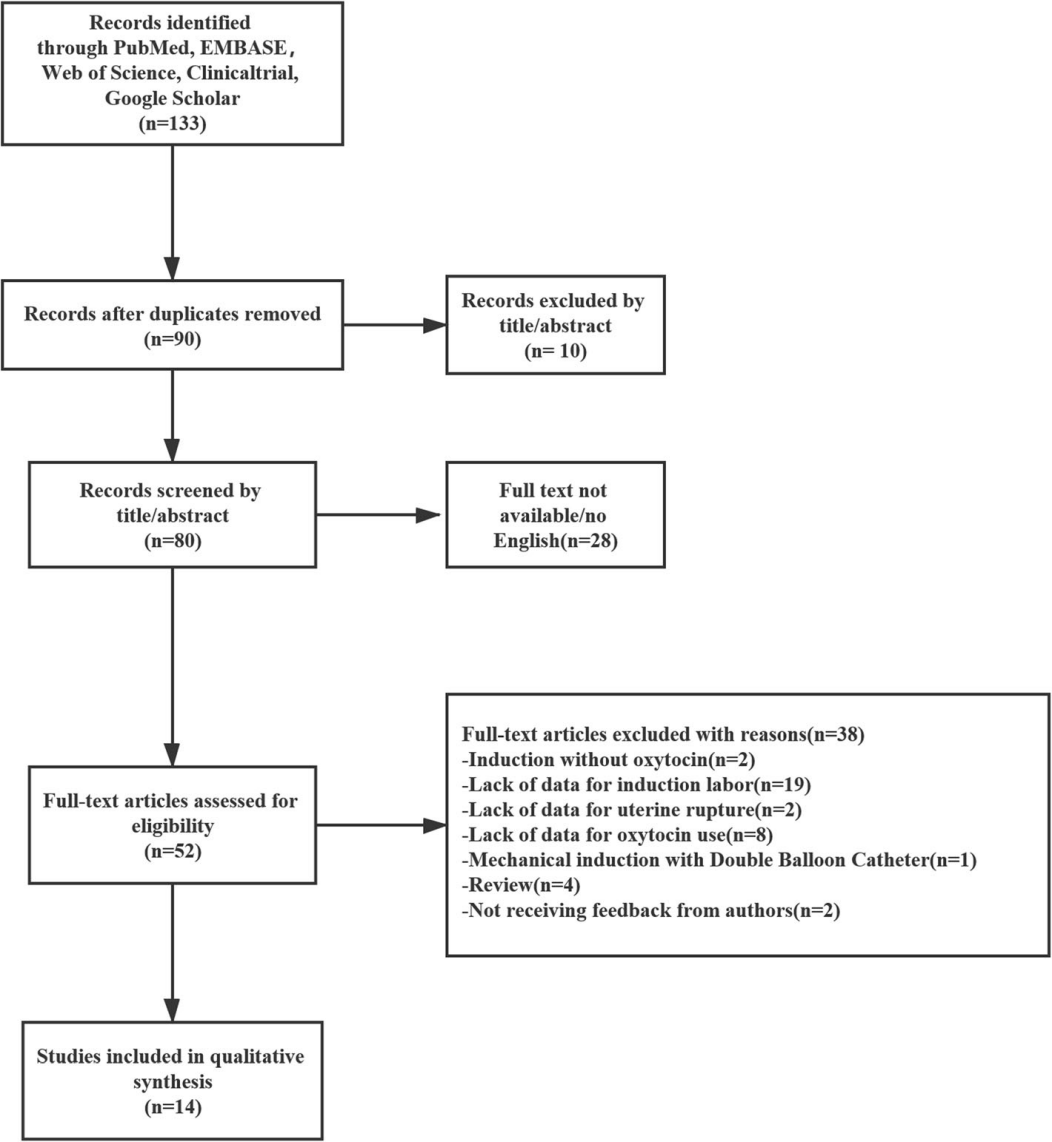
shows the screening process of this meta-analysis. According to the including criteria, a literature search was conducted through. Finally, 14 relevent studies were included after screening

A total of 14 studies, which included 48,457 women who underwent TOLAC, met the inclusion criteria. The summary of the screening process is presented in Fig. 1. A total of 36,596 women had spontaneous delivery, and 11,861 had induced labor. In the spontaneous delivery group, 11,969 women had successful VBAC, 9823 women used oxytocin augmentation, and 223 women presented with uterine rupture. Among those with induced labor, 3195 had successful VBAC, 5148 used oxytocin induction, and 41 presented with uterine rupture in the induction of labor. In addition, in 11 studies, the uterine rupture was clearly described, while it was unclear in 3 studies [24–26]. Other demographic and clinical information for each study are shown in Table 1. The rates of VBAC, oxytocin use and uterine rupture in women with spontaneous and induction labor were listed in Table 2. In addition, the methodological quality of the studies assessed by NOS is presented in the Supplement materials. Most included studies were scored with six or more stars, representing high quality.

**Table 1:** Characteristis of the included studies in this meta-analysis

Study	Year	Spontaneous labor							Induction labor							Initial oxytocin dose	Interval to dose increase	Maximum dose
		N (oxytocin augmentation)	VBAC	Uterine rupture	Maternal age(y)	Maternal BMI	Gestational age (weeks)	Parity	N (oxytocin induction)	VBAC	Uterine rupture	Maternal age(y)	Maternal BMI	Gestational age (weeks)	Parity			
Fishel-Bartal [24]	2019	723 (46)	590	7	34.1	28.2	39	1.8	107 (107)	81	2 (2)	33.8	29.1	> 34	1.71	2 miU/min	20 min	
Gobillot [27]	2018	553							248 (248)	145	8 (8)	31.64	25.56	39.41	1.51	2.5 miU/min	30 min	20miU
Kiwan [25]	2018	477 (218)	318	0	31.1		37.89	1.9	90 (45)	45	1	30.84		39.89	2.2			
Hehir [26]	2016	2222 (235)	1611	4	32.1		> 37		849 (370)	370						5 mU/min	15 min	30 mU/min
Stenson [28]	2015	1193	749	0					208 (107)	107	9 (7)	34.23	25.42					
Ashwal [29]	2014	1639 (1291)	1258	19	35.32		39.17	3	259 (186)	186	3	35.28		39.28	3	2.5 mU/min	20 min	
Shatz [30]	2013	4263	3111	6	29.18				1576 (1062)	1062	9	29.13						
Ouzounian [31]	2011	5026 (53)	4332	52	29.7		39.4	1.6	1806 (1199)	1199	22	30		40.4	1.6			
Kwee [32]	2007	2592 (536)		27 (10)					682 (308)		4							
Lin [33]	2004	2523		11					572 (430)		7 (5)							
Landon [6]	2004	12,694 (6009)		76 (52)	28.7				4708 (1864)		48	28.7						
Blanchette [34]	2001	292 (288)		5 (4)					174 (174)		7 (7)							
Zelop [13]	1999	2214 (1072)		16 (11)					560		13 (12)					1 to 2 mU/min to 20 mU/min		
Chua [35]	1989	185 (75)		0					22 (22)		0					2.5 mU/min	30 min	12.5 mU/min

*VBAC* Vaginal birth after cesarean

**Table 2:** The rates of VBAC, oxytocin use and uterine rupture in women with spontaneous and induction labor

Study	Year	Spontaneous labor			Induction labor		
		Rate of VBAC	Rate of oxytocin use	Rate of uterine rupture	Rate of VBAC	Rate of oxytocin use	Rate of uterine rupture
Fishel-Bartal [24]	2019	0.816	0.064	0.010	0.757	1	0.019
Gobillot [27]	2018	–	–	–	0.585	1	0.032
Kiwan [25]	2018	0.667	0.457	–	0.500	–	–
Stenson [28]	2015	0.628	–	–	0.514	0.178	0.034
Ashwal [29]	2014	0.768	0.788	0.012	0.718	0.490	–
Shatz [30]	2013	0.730	–	0.001	0.674	–	–
Ouzounian [31]	2011	0.862	0.011	0.010	0.664	0.690	–
Kwee [32]	2007	–	0.207	0.010	–	0.452	–
Landon [6]	2004	–	0.473	0.006	–	0.752	0.009
Blanchette [34]	2001	–	0.986	0.017	–	1	0.040
Zelop [13]	1999	–	0.484	0.007	–	1	–

*VBAC* Vaginal birth after cesarean

### Differences in pooled rates between spontaneous delivery and induction of labor

The pooled usage rate of oxytocin, rate of VBAC and rate of uterine rupture in spontaneous delivery group were 39.8% (95%CI: 0.532 to 0.682; *p* = 0.001; P_heterogeneity_ < 0.001), 74.3% (95%CI: 0.679 to 0.807; *p* = 0.001; P_heterogeneity_ < 0.001) and 0.7% (95%CI: 0.004 to 0.009; *p* < 0.001; P_heterogeneity_ < 0.001), respectively; while in induced labor group were 60.6% (95%CI: 0.452 to 0.759; *p* < 0.001; P_heterogeneity_ < 0.001), 60.7% (95%CI: 0.532 to 0.682; *p* < 0.001; P_heterogeneity_ < 0.001) and 2.2% (95%CI: 0.012 to 0.033; *p* = 0.0001; P_heterogeneity_ = 0.051) respectively. These results suggested that the women with spontaneous delivery had significantly higher rate of VBAC (Z = 3.43; *p* = 0.001), and lower rate of uterine rupture (Z = 2.96; *p* = 0.003) than those who underwent induced labor, while there was no significant difference in oxytocin usage rate (Z = -1.4797; *p* = 0.0805; (see Supplementary materials, Fig. S1, S2, S3).

To explore possible sources of heterogeneity, we calculated the synthetic effect size in the spontaneous delivery group and induced labor group; 3 studies which did not have a clear definition of uterine rupture were excluded [24–26]. The pooled usage rate of oxytocin, rate of VBAC and rate of uterine rupture in spontaneous delivery were 74.7% (95%CI: 0.654 to 0.84; *p* < 0.0001; P_heterogeneity_ < 0.001), 47.9% (95%CI: 0.165 to 0.793; *p* = 0.003; P_heterogeneity_ < 0.001) and 0.7% (95%CI: 0.004 to 0.010; *p* < 0.001; P_heterogeneity_ < 0.001), respectively; while in induction of labor were 63.8% (95%CI: 0.593 to 0.684; *p* < 0.001; P_heterogeneity_ < 0.001), 55.7% (95%CI: 0.360 to 0.754; *p* < 0.001; P_heterogeneity_ < 0.001) and 2.3% (95%CI: 0.011 to 0.035; *p* < 0.001; P_heterogeneity_ = 0.027), respectively. However, heterogeneity obviously increased after re-analyzing.

To assess the influence of outliers, the standardized residual was examined for all studies. We conducted the sensitivity analysis by removing one study at the time to evaluate the weights of individual studies on the pooled SMDs. Fig. S4 showed that sensitivity analysis was relatively robust for the meta-analysis, with no reverse outcomes (see Supplementary materials).

Funnel plots and Egger’s test were used to reveal possible publication bias. The results showed a possible overestimation of effect size in the usage rate of oxytocin in spontaneous delivery (*p* = 0.048), the rate of uterine rupture in both spontaneous delivery (*p* = 0.031) and induction of labor (*p* = 0.019; see Supplementary materials**,** Fig. S5).

Despite high methodological quality, direct evidence, and precision, the included studies were characterized by moderate heterogeneity, thus providing the evidence to understand the way of moderate quality (GRADE rating system) (see Supplementary materials).

### Differences in rates of uterine rupture in using oxytocin and not using oxytocin in TOLAC

**Fig. 2:**
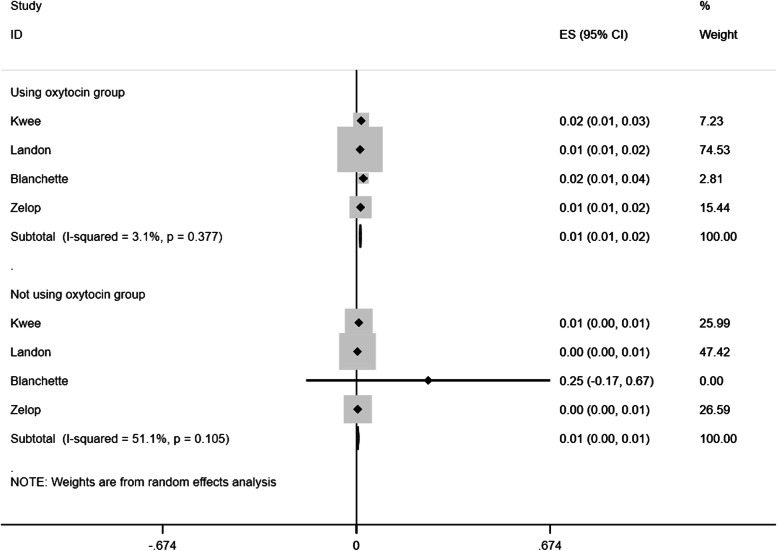
The pooled rate in women using oxytocin in TOLAC was 1.4%(95%CI: 0.011 to 0.016; *p* < 0.001) and that in women not using oxytocin in TOLAC was 0.5%(95%CI: 0.002 to 0.008; *p* < 0.001). TOLAC, Trial of labor after a previous cesarean delivery; ES, effect size; CI, confidence interval

**Fig. 3:**
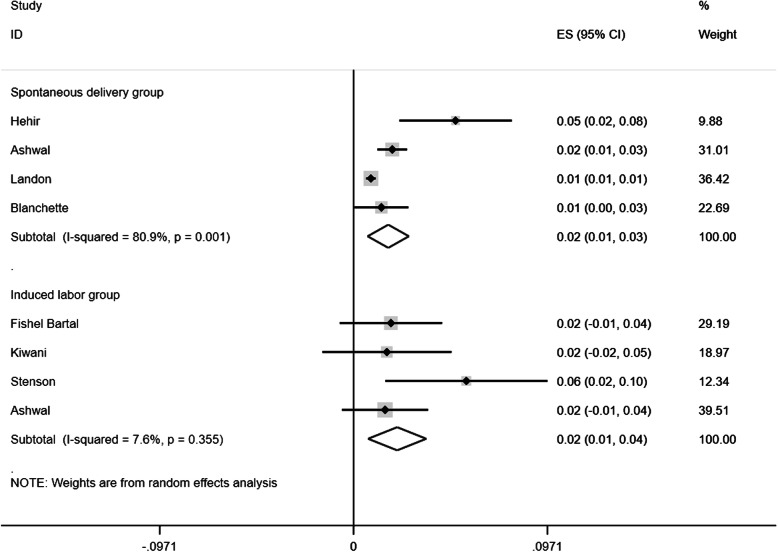
The pooled rates of uterine rupture in oxytocin augmentation among women with spontaneous labor and those with induction of labor were 1.7% (95%CI: 0.007 to 0.028; *p* = 0.001) and 2.2% (95%CI: 0.007 to 0.036; *p* = 0.003), respectively. ES, effect size; CI, confidence interval

The pooled rates of uterine rupture in women who were using oxytocin and those who were not in TOLAC were 1.4% (95%CI: 0.011 to 0.016; *p* < 0.001; P_heterogeneity_ = 0.377) and 0.5% (95%CI: 0.002 to 0.008; *p* < 0.001; P_heterogeneity_ = 0.105, Fig. 2), respectively, and the difference was statistically significant (Z = 7.3259; *p* = 0.0002). On the basis of the previous results, we calculated the pooled rates of uterine rupture in oxytocin augmentation among women with spontaneous labor and those with induction of labor, which were 1.7% (95%CI: 0.007 to 0.028; *p* = 0.001; P_heterogeneity_ = 0.0.001) and 2.2% (95%CI: 0.007 to 0.036; *p* = 0.003; P_heterogeneity_ = 0.355, Fig. 3), respectively, and the difference was not significant (Z = -0.77; *p* = 0.443).

## Discussion

The primary purpose of the current meta-analysis was to systematically identify the effect of oxytocin on the risk of uterine rupture in TOLAC. In addition, we also explored differences in the usage rate of oxytocin, rate of VBAC between spontaneous delivery and induction of labor. In this study, we identified 14 studies, which included 48,457 women undergoing TOLAC. The results showed that women with spontaneous delivery had a higher rate of VBAC and lower risk of uterine rupture than those with the induced labor. The risk of uterine rupture increased in women using oxytocin in TOLAC compared to those not using oxytocin in TOLAC. Moreover, we compared the risk of uterine rupture in augmentation among women with spontaneous labor and women who had a successful induction of labor whilst controlling for potential factors, which revealed no significant statistical differences. These data revealed that the process labor induction or cervix condition might influence the effect of oxytocin on the uterine.

In our study, the VBAC rates in women with spontaneous delivery and labor induction were 74.3 and 60.7%, respectively. These results were consistent with a previous report, which suggested that the average success rate of a TOLAC resulting in a VBAC ranges between 60 and 80% [36–40]. We also found that the VBAC rate was significantly different between women with spontaneous and induction labor. Some mechanisms, such as inflammatory cytokines, regulation of endocrine hormone, and mechanical stimulus, may contribute to these differences [41]. Besides, we found that the rates of oxytocin usage in women with spontaneous and induction labor were 39.8 and 60.6%, respectively, though there was no significant difference between groups. Other prognostic variables, including maternal age < 40 years, ethnicity, body mass index (BMI) < 30, gestational age < 40 weeks, infant birth weight < 4 kg, and higher admission bishop score might also influence the results [42, 43]. However, we were unable to obtain enough information to separately analyze these variables.

Our results demonstrated a low rate of uterine rupture in women attempting TOLAC with spontaneous versus induced labor (0.7% versus 2.2%, *p* = 0.0003). A previous study found that the rate of uterine rupture was from 0.15 to 5.5% in women with spontaneous labor and from 0.3 to 10.7% in women with induced labor [44]. Thus, we concluded that the rate of uterine rupture tends to vary from study to study, which may be partly due to the way uterine rupture is defined in researches. Generally speaking, uterine rupture includes complete uterine rupture and incomplete rupture (uterine scar dehiscence). This study took a definition of complete uterine rupture. However, incomplete uterine rupture is defined as a disruption of the uterine muscle with intact uterine serosa, which is mostly asymptomatic. Different conditions may lead to different outcomes. Complete uterine rupture is associated with severe maternal (risk of hemorrhage with a need for transfusion, hysterectomy) and perinatal (risks of anoxoischemic encephalopathy, and even fetal death) morbidity and mortality. In addition, it remains one of the most catastrophic obstetrical emergencies [45]. However, uterine dehiscence has little to no consequence on immediate maternal or neonatal morbidity and is only detected in cases of emergency cesarean section [46]. To avoid the effect of ambiguous definition on heterogeneity, we have calculated the pooled effect by excluding the studies that did not have a clear explanation of uterine rupture. The results showed that heterogeneity obviously increased, which proved that heterogeneity did not come from the ambiguous definition. Moreover, an unavoidable source of heterogeneity may come from the medical level in different areas. In a general way, senior or specialized hospitals may have more experience and the ability to prevent and treat a uterine rupture. In contrast, junior or comprehensive hospitals may have a higher rate of uterine rupture. It may cause the main heterogeneity of these results, but we need more detailed information to deal with this issue.

The most exciting aspect of our study was the safety of oxytocin using in TOLAC. Consequently, we compared the rate of uterine rupture in women using oxytocin and not using oxytocin in TOLAC (1.4% versus 0.5%). We found that oxytocin may increase the risk of uterine rupture in TOLAC. According to the previous researches, the association between oxytocin and uterine rupture remains unknown. In Goetzl’s study, no difference in oxytocin duration or oxytocin dose was found between cases of uterine rupture and controls [47]. Contrary, Landon et al reported that augmentation and induction with oxytocin were associated with an increased risk of uterine rupture [6]. In the studies of Cahill et al, there was a dose-response relationship between maximum oxytocin dose and risk of uterine rupture. They argued that higher maximum dose of oxytocin should be cautiously used in a trial of labor after cesarean and that an upper limit of oxytocin in TOLAC should be 20 mU/min [48, 49], which suggested that long-time exposure to oxytocin in TOLAC is positively correlated with higher risk of uterine rupture. This provided an explanation for why women who had more labors induced with oxytocin were at greater risk of uterine rupture than those with spontaneous labor.

To further investigate how oxytocin increases the risk of uterine rupture, we compared the risk of uterine rupture in labor augmentation among women with spontaneous labor and women with successfully induced labor. We found no statistical significance between groups, which supported the premise that the process of induction labor or individual cervix condition may change the effect of oxytocin and increase the risk of uterine rupture. Previous researches reported that other potential factors, such as induction of labor with prostaglandin or Foley catheters, could also increase the risk of uterine rupture compared to spontaneous delivery [50, 51]. The process of induction may make scarred uteruses more sensitive and brittle. Our study also showed that neither oxytocin nor other way of induction could indirectly increase postpartum complications; however, this needs to be further investigated by future studies. Another possible cause of increased risk may be the individual cervix condition. Unfavorable cervix could enhance the difficulty of parturition, which in turn could increase the chances of exposure to risk factors. The Bishop score is commonly used in most clinical evaluations for the ripeness of the cervix. A previous study reported that women who underwent induction with a favorable cervical score had a lower rate of uterine rupture [52]. While this assessment tool is not perfect in its repeatability and objectivity, currently it is the best option [53]. More suitable evaluation tools need to be developed to help clinical observation.

### Limitations

The present meta-analysis has some limitations. First, we could only make the conclusion on the risk association between oxytocin and uterine rupture. However, different protocols of oxytocin use in TOLAC may lead to different outcomes. Thus, more studies are needed in the future. Second, the high heterogeneity among the included studies could not be ignored. Though we have excluded the influence of the diagnosis, other potential factors, such as maternal age, ethnicity, BMI, gestational age, infant birth weight, higher admission bishop score, medical level, and so on could also be relevant. However, it was not possible to obtain more detailed information from the included studies. Though it is common for meta-analyses of observational studies to present high heterogeneity, more studies in the future are necessary. In addition, we have made the Newcastle-Ottawa Scale to evaluate and ensure the quality of the included studies so as to reduce bias as much as possible.

## Conclusion

Overall, our study has demonstrated the risk association between oxytocin use in TOLAC and uterine rupture. We also found that the process of induction or cervix condition might influence the effect of oxytocin on uteruses with a scar. Based on our results, simplified and standardized intrapartum management and cautiously monitoring of oxytocin use could help to avoid some maternal and neonatal complications [54]. On the other hand, more studies are needed to explore how oxytocin affects the process of TOLAC. It is necessary to consider precise initial dose, maximum dose, the interval to dose increase, and duration of oxytocin to reduce possible risk and enhance the safety of TOLAC.

## Supplementary Information

**Additional file 1: Fig. S1**. The pooled usage rate of oxytocin in spontaneous delivery group and induced labor group were 39.8% (95%CI: 0.532 to 0.682; *p* = 0.001; Pheterogeneity < 0.001) and 60.6% (95%CI: 0.452 to 0.759; *p* < 0.001; Pheterogeneity < 0.001), respectively. TOLAC, Trial of labor after a previous cesarean delivery; ES, effect size; CI, confidence interval.

**Additional file 2: Fig. S2**. The pooled rate of VBAC in spontaneous delivery group and induced labor group were 74.3% (95%CI: 0.679 to 0.807; *p* = 0.001; Pheterogeneity < 0.001) and 60.7% (95%CI: 0.532 to 0.682; *p* < 0.001; Pheterogeneity < 0.001), respectively. VBAC, vaginal birth after a cesarean section; ES, effect size; CI, confidence interval.

**Additional file 3: Fig. S3**. The pooled rate of uterine rupture in spontaneous delivery group and induced labor group were 0.7% (95%CI: 0.004 to 0.009; *p* < 0.001; Pheterogeneity < 0.001) and 2.2% (95%CI: 0.012 to 0.033; *p* = 0.0001; Pheterogeneity =0.051) respectively. TOLAC, Trial of labor after a previous cesarean delivery; ES, effect size; CI, confidence interval.

**Additional file 4: Fig. S4**. The sensitivity analysis by removing one study at the time to evaluate the weights of individual studies on the pooled SMDs. The result was relatively robust for the meta-analysis, with no reverse outcomes.

**Additional file 5: Fig. S5**. Funnel plots and Egger’s test were used to reveal possible publication bias. The results showed a possible overestimation of effect size in the usage rate of oxytocin in spontaneous delivery (*p* = 0.048), the rate of uterine rupture in both spontaneous delivery (*p* = 0.031) and induction of labor.

**Additional file 6.**

**Additional file 7.**

**Additional file 8.**

## Data Availability

Not applicable.

## Abbreviations

TOLAC: Trial of labor after a previous cesarean delivery
CS: Cesarean section
VBAC: Vaginal birth after a cesarean section
ACOG: The American College of obstetricians and gynecologists
PRISMA: The preferred reporting items for systematic reviews and meta-analyses
NOS: The Newcastle-Ottawa scale
GRADE: The grading of recommendations assessment, development and evaluation
CI: Confidence interval
