# Supplementary figures and images for: Oxytocin use in trial of labor after cesarean and its relationship with risk of uterine rupture in women with one previous cesarean section: a meta-analysis of observational studies
Source: BMC Pregnancy Childbirth. 2021 Jan 6;21:11. doi: 10.1186/s12884-020-03440-7 (PMC7786988; doi:10.1186/s12884-020-03440-7)

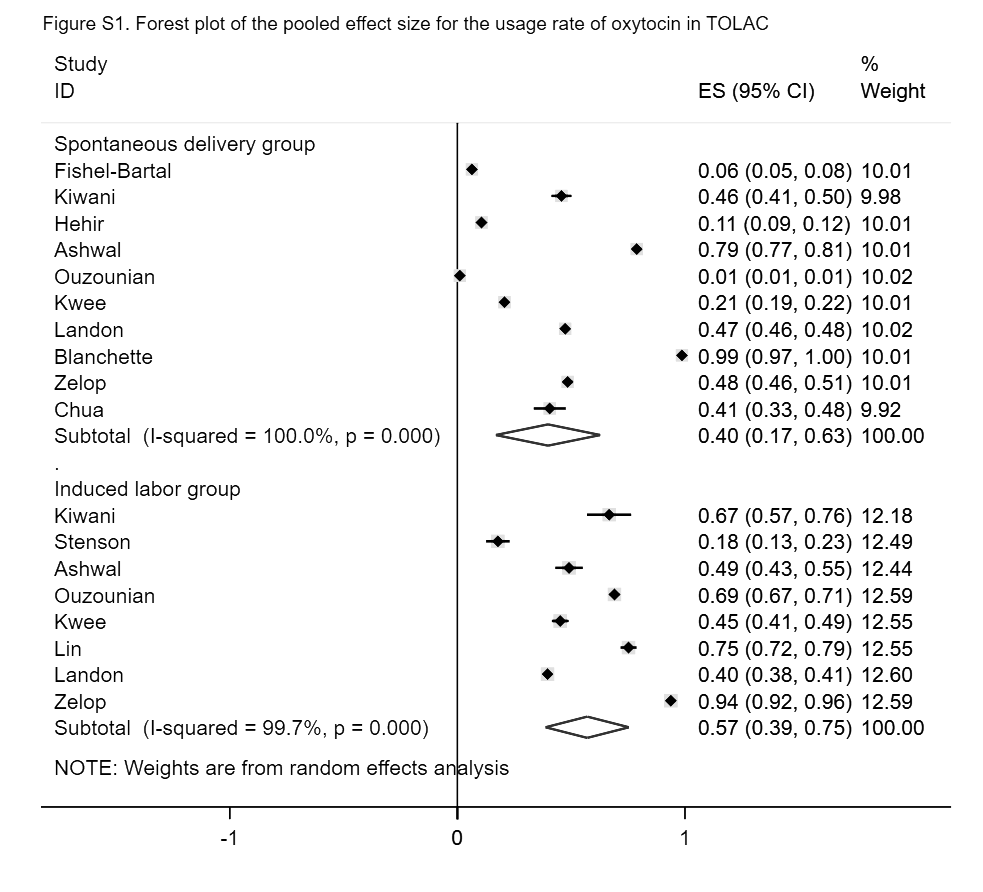

Supplement: Supplementary file 1 — Additional file 1: Fig. S1. The pooled usage rate of oxytocin in spontaneous delivery group and induced labor group were 39.8% (95%CI: 0.532 to 0.682; p = 0.001; Pheterogeneity < 0.001) and 60.6% (95%CI: 0.452 to 0.759; p < 0.001; Pheterogeneity < 0.001), respectively. TOLAC, Trial of labor after a previous cesarean delivery; ES, effect size; CI, confidence interval. [file 12884_2020_3440_MOESM1_ESM.zip › Fig.S1_revision.png]

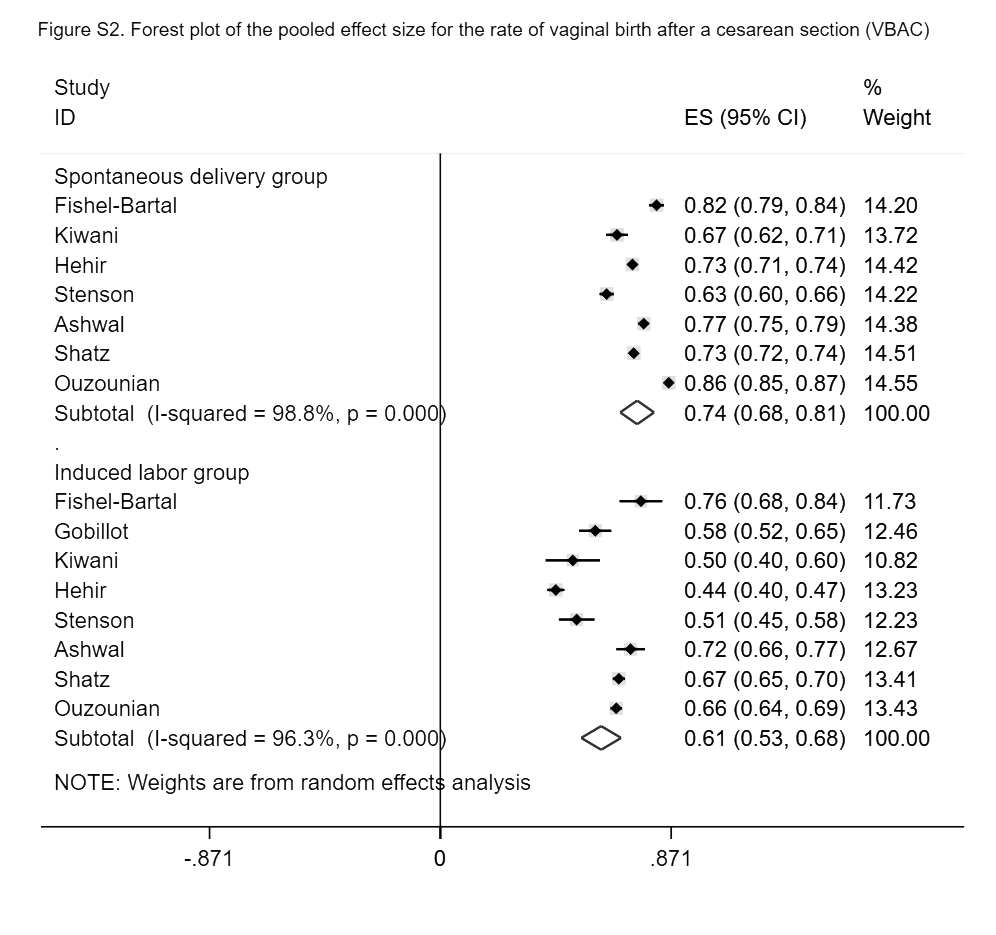

Supplement: Supplementary file 2 — Additional file 2: Fig. S2. The pooled rate of VBAC in spontaneous delivery group and induced labor group were 74.3% (95%CI: 0.679 to 0.807; p = 0.001; Pheterogeneity < 0.001) and 60.7% (95%CI: 0.532 to 0.682; p < 0.001; Pheterogeneity < 0.001), respectively. VBAC, vaginal birth after a cesarean section; ES, effect size; CI, confidence interval. [file 12884_2020_3440_MOESM2_ESM.zip › Fig.S2_revision.png]

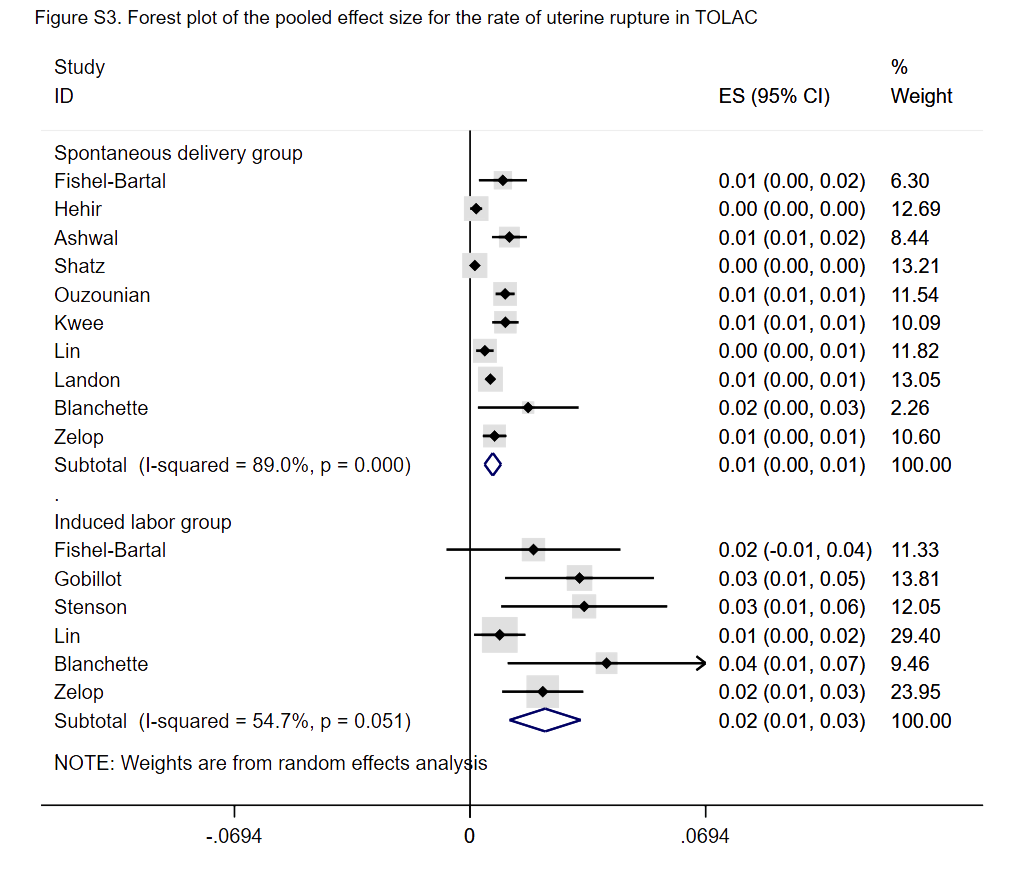

Supplement: Supplementary file 3 — Additional file 3: Fig. S3. The pooled rate of uterine rupture in spontaneous delivery group and induced labor group were 0.7% (95%CI: 0.004 to 0.009; p < 0.001; Pheterogeneity < 0.001) and 2.2% (95%CI: 0.012 to 0.033; p = 0.0001; Pheterogeneity =0.051) respectively. TOLAC, Trial of labor after a previous cesarean delivery; ES, effect size; CI, confidence interval. [file 12884_2020_3440_MOESM3_ESM.zip › Fig.S3_revision.png]

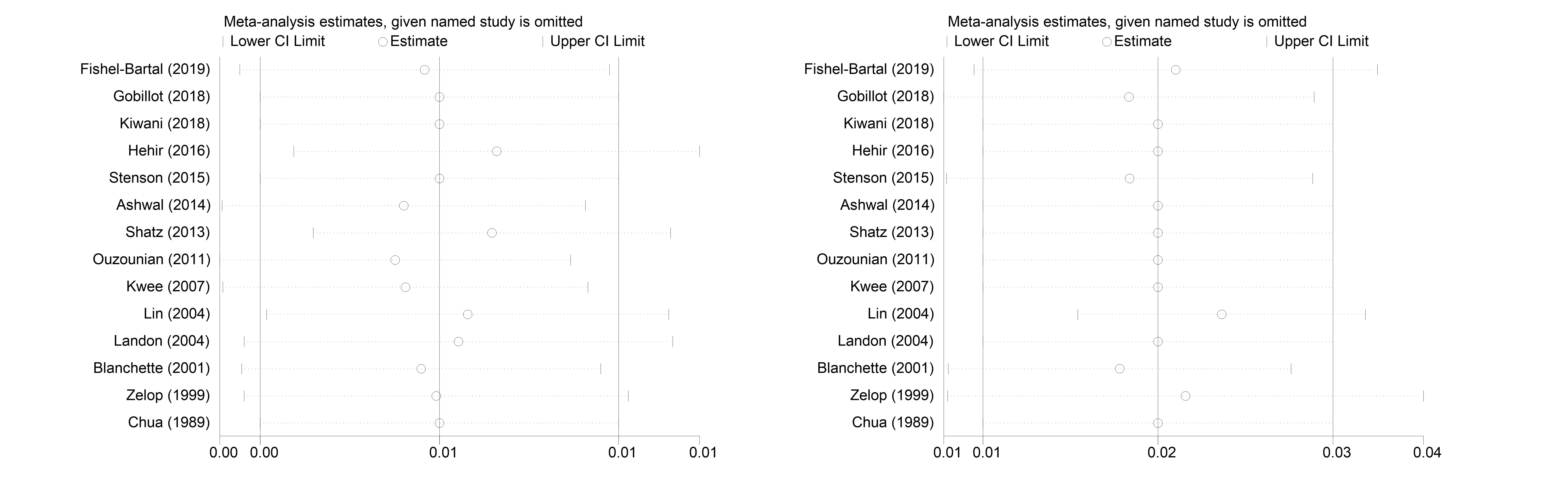

Supplement: Supplementary file 4 — Additional file 4: Fig. S4. The sensitivity analysis by removing one study at the time to evaluate the weights of individual studies on the pooled SMDs. The result was relatively robust for the meta-analysis, with no reverse outcomes. [file 12884_2020_3440_MOESM4_ESM.zip › S4_revision.png]

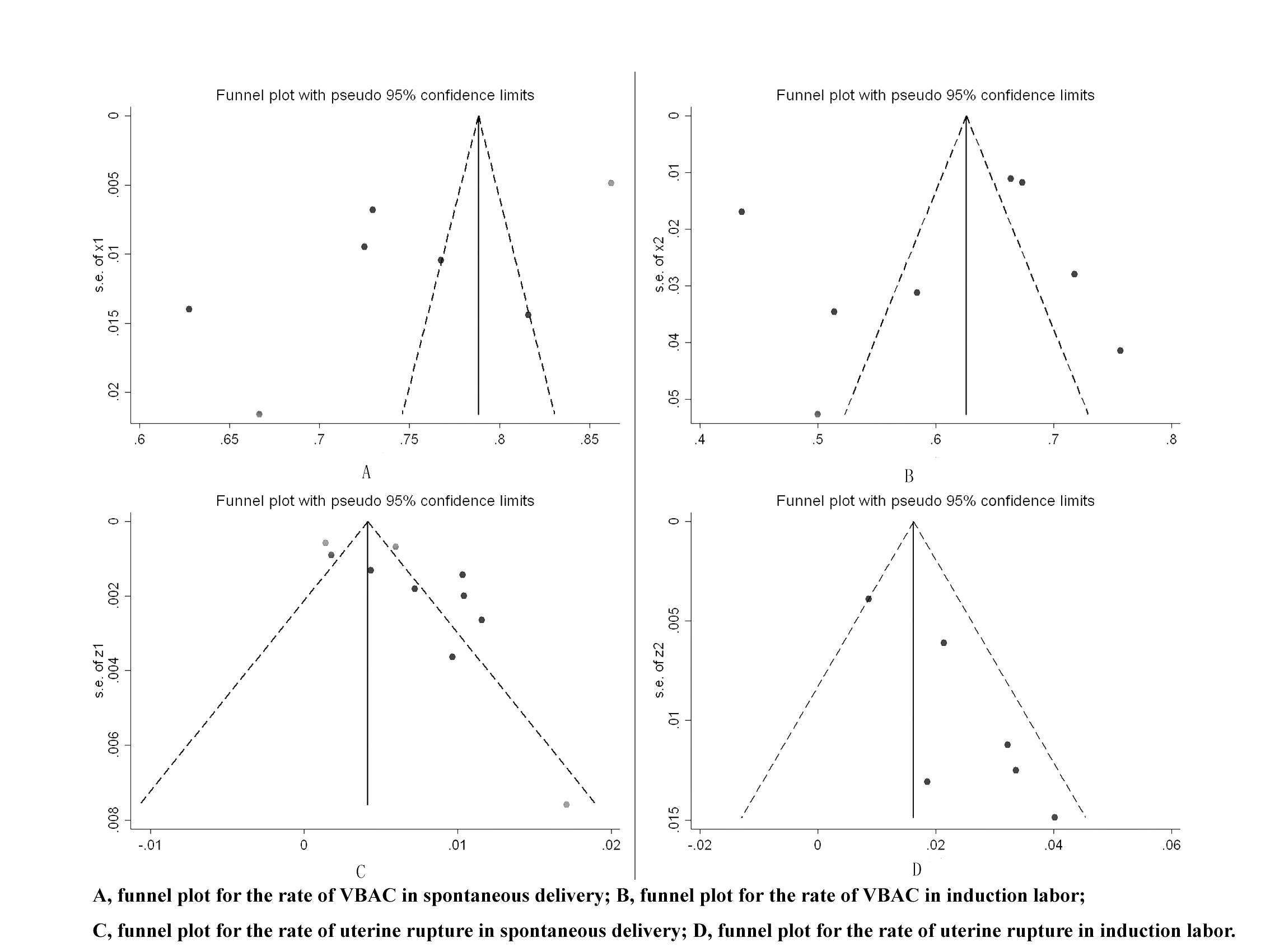

Supplement: Supplementary file 5 — Additional file 5: Fig. S5. Funnel plots and Egger’s test were used to reveal possible publication bias. The results showed a possible overestimation of effect size in the usage rate of oxytocin in spontaneous delivery (p = 0.048), the rate of uterine rupture in both spontaneous delivery (p = 0.031) and induction of labor. [file 12884_2020_3440_MOESM5_ESM.zip › Figure S5_revision.png]
