# Supplementary material for: Oxytocin use in trial of labor after cesarean and its relationship with risk of uterine rupture in women with one previous cesarean section: a meta-analysis of observational studies
Source: BMC Pregnancy Childbirth. 2021 Jan 6;21:11. doi: 10.1186/s12884-020-03440-7 (PMC7786988; doi:10.1186/s12884-020-03440-7)
Supplement: Supplementary file 6 — Additional file 6. [file 12884_2020_3440_MOESM6_ESM.docx]

**Search strategy**

The detailed search strategy used in the Pubmed was “((((((((((("vagina"[MeSH Terms] OR "vagina"[All Fields]) OR "vaginal"[All Fields]) OR "vaginally"[All Fields]) OR "vaginals"[All Fields]) OR "vaginitis"[MeSH Terms]) OR "vaginitis"[All Fields]) OR "vaginitides"[All Fields]) AND (((((("birth s"[All Fields] OR "birthed"[All Fields]) OR "birthing"[All Fields]) OR "parturition"[MeSH Terms]) OR "parturition"[All Fields]) OR "birth"[All Fields]) OR "births"[All Fields]) AND "after"[All Fields] AND ((("caesarean section"[All Fields] OR "cesarean section"[MeSH Terms]) OR ("cesarean"[All Fields] AND "section"[All Fields])) OR "cesarean section"[All Fields])) OR ((("vaginal birth after cesarean"[MeSH Terms] OR ((("vaginal"[All Fields] AND "birth"[All Fields]) AND "after"[All Fields]) AND "cesarean"[All Fields])) OR "vaginal birth after cesarean"[All Fields]) OR "vbac"[All Fields])) AND (((((("trial of labour"[All Fields] OR "trial of labor"[MeSH Terms]) OR ("trial"[All Fields] AND "labor"[All Fields])) OR "trial of labor"[All Fields]) AND "after"[All Fields] AND ((("caesarean section"[All Fields] OR "cesarean section"[MeSH Terms]) OR ("cesarean"[All Fields] AND "section"[All Fields])) OR "cesarean section"[All Fields])) OR ((("trial of labour"[All Fields] OR "trial of labor"[MeSH Terms]) OR ("trial"[All Fields] AND "labor"[All Fields])) OR "trial of labor"[All Fields])) OR ("tolac"[All Fields] OR "tolacs"[All Fields]))) AND (((((("oxytocin"[MeSH Terms] OR "oxytocin"[All Fields]) OR "oxytocin s"[All Fields]) OR "oxytocine"[All Fields]) OR "oxytocins"[All Fields]) OR ((((("oxytocin"[MeSH Terms] OR "oxytocin"[All Fields]) OR "oxytocin s"[All Fields]) OR "oxytocine"[All Fields]) OR "oxytocins"[All Fields]) AND ((((((((("infusate"[All Fields] OR "infusates"[All Fields]) OR "infuse"[All Fields]) OR "infused"[All Fields]) OR "infuser"[All Fields]) OR "infusers"[All Fields]) OR "infuses"[All Fields]) OR "infusing"[All Fields]) OR "infusion"[All Fields]) OR "infusions"[All Fields]))) OR (((("labor, induced"[MeSH Terms] OR ("labor"[All Fields] AND "induced"[All Fields])) OR "induced labor"[All Fields]) OR ("induction"[All Fields] AND "labor"[All Fields])) OR "induction of labor"[All Fields]))) AND ((("uterine rupture"[MeSH Terms] OR ("uterine"[All Fields] AND "rupture"[All Fields])) OR "uterine rupture"[All Fields]) OR (((((("maternally"[All Fields] OR "maternities"[All Fields]) OR "maternity"[All Fields]) OR "mothers"[MeSH Terms]) OR "mothers"[All Fields]) OR "maternal"[All Fields]) AND (((((("epidemiology"[MeSH Subheading] OR "epidemiology"[All Fields]) OR "morbidity"[All Fields]) OR "morbidity"[MeSH Terms]) OR "morbid"[All Fields]) OR "morbidities"[All Fields]) OR "morbids"[All Fields]))).”
