# Supplementary material for: Oxytocin use in trial of labor after cesarean and its relationship with risk of uterine rupture in women with one previous cesarean section: a meta-analysis of observational studies
Source: BMC Pregnancy Childbirth. 2021 Jan 6;21:11. doi: 10.1186/s12884-020-03440-7 (PMC7786988; doi:10.1186/s12884-020-03440-7)
Supplement: Supplementary file 7 — Additional file 7. [file 12884_2020_3440_MOESM7_ESM.docx]

**Evaluation of the Grading of Recommendations Assessment, Development and Evaluation (GRADE) certainty ratings system.**

| **people with substance dependence or chronic substance heavy users compared to non-substance users or limited users for risky decision-making performance** | | | | | |
| --- | --- | --- | --- | --- | --- |
| **Patient or population:** people with substance dependence or chronic substance heavy users  **Comparison:** non-substance users or limited users **Intervention:** risky decision-making performance   \| **Quality assessment** \| \| \| \| \| \| \| **No of patients** \| \| **Effect Size** \| \| **Quality** \| **Importance** \|  \| \| --- \| --- \| --- \| --- \| --- \| --- \| --- \| --- \| --- \| --- \| --- \| --- \| --- \| --- \| \|  \| \| **No of studies** \| **Design** \| **Risk of bias** \| **Inconsistency** \| **Indirectness** \| **Imprecision** \| **Other considerations** \| **People with substance dependence or chronic substance heavy users** \| **Non-substance users or limited users** \| **Relative  (95% CI)** \| **SMD** \|  \| \| **Risky decision-making performance** \| \| \| \| \| \| \| \| \| \| \| \| \|  \| \| 14 \| observational studies^1^ \| no serious risk of bias \| serious^1^ \| no serious indirectness \| no serious imprecision \| reporting bias^1^ \| 48457 women undergoing TOLAC \| \|  \| 0.238 \| ⊕OOO  VERY LOW \| CRITICAL \|  \| \|  \|  \|  \|  \|  \|  \|  \|  \|  \|  \|  \|  \|  \|  \| | | | | | |
| **Outcomes** | **Illustrative comparative risks* (95% CI)** | | **No of Participants (studies)** | **Quality of the evidence (GRADE)** |  |
|  | Assumed risk | Corresponding risk |  |  |  |
|  | **women having spontaneous delivery** | **women having induction of labor** |  |  |  |
| **Rate of uterine rupture** | 0.7% (95%CI: 0.004 to 0.009; p<0.001) | 2.2% (95%CI: 0.012 to 0.033; p=0.0001) | Total of 36596 women having spontaneous delivery and 11861 women having induction of labor (14 studies) | ⊕⊝⊝⊝ **low**^1^ |  |
| *The basis for the **assumed risk** (e.g. the median control group risk across studies) is provided in footnotes. The **corresponding risk** (and its 95% confidence interval) is based on the assumed risk in the comparison group and the **relative effect** of the intervention (and its 95% CI). | | | | | |
| **CI:** Confidence interval; **OR:** Odds ratio; | | | | | |
| GRADE Working Group grades of evidence **High quality:** Further research is very unlikely to change our confidence in the estimate of effect.  **Moderate quality:** Further research is likely to have an important impact on our confidence in the estimate of effect and may change the estimate. **Low quality:** Further research is very likely to have an important impact on our confidence in the estimate of effect and is likely to change the estimate. **Very low quality:** We are very uncertain about the estimate. | | | | | |
| ^1^ observational studies | | | | | |
