# Supplementary material for: Oxytocin use in trial of labor after cesarean and its relationship with risk of uterine rupture in women with one previous cesarean section: a meta-analysis of observational studies
Source: BMC Pregnancy Childbirth. 2021 Jan 6;21:11. doi: 10.1186/s12884-020-03440-7 (PMC7786988; doi:10.1186/s12884-020-03440-7)
Supplement: Supplementary file 8 — Additional file 8. [file 12884_2020_3440_MOESM8_ESM.docx]

**Evaluation of Quality of the Studies According to the Newcastle-Ottawa Scale**

| **Study** | **Selection** | | | | **Comparability** | **Exposure** | | | | **Scores** |
| --- | --- | --- | --- | --- | --- | --- | --- | --- | --- | --- |
|  | **1A** | **2A** | **3A** | **4A** | **5A** | | **6A** | **7A** | **8A** |  |
| Fishel Bartal,2019 | * |  | * | * | ** | | * | * |  | 7 |
| Kiwani,2018 | * | * | * | * | * | | * | * |  | 7 |
| Gobillot,2018 | * | * | * | * | * | | * | * |  | 7 |
| Hehir,2016 |  |  | * | * | ** | | * | * |  | 6 |
| Stenson,2015 | * | * | * | * | ** | | * | * |  | 8 |
| Ashwal,2014 | * | * | * | * | ** | | * | * |  | 8 |
| Shatz,2013 | * | * | * | * | ** | | * | * |  | 8 |
| Ouzounian,2011 | * | * | * | * |  | | * | * |  | 6 |
| Kwee,2007 | * | * | * | * | ** | | * | * |  | 8 |
| Landon,2004 | * | * | * | * | * | | * | * |  | 7 |
| Lin,2004 | * | * | * | * | * | | * | * |  | 7 |
| Blanchette,2001 | * | * | * | * |  | | * | * |  | 6 |
| Zelop,1999 | * | * | * | * | * | | * | * |  | 7 |
| Chua,1989 | * | * | * | * | * | | * | * |  | 7 |

Asterisks indicate that the quality item is present. A study can be awarded a maximum of one asterisk for each numbered item within the selection and exposure or outcome categories. A maximum of two asterisks can be given for comparability (i.e., study controls for the most important factor and any additional factor). The total number of asterisks represents the final score.

1A = case definition was adequate, 2A = representativeness of the cases; 3A = selection of controls, 4A = definition of controls; 5A = comparability of cases and controls on the basis of analysis; 6A = ascertainment of exposure; 7A = same method of ascertainment for cases and controls; 8A = nonresponse rate.

Scores ≥ 6 (range 0–9) indicate high-quality studies.
